# Translational tests involving non-reward: methodological considerations

**DOI:** 10.1007/s00213-018-5062-x

**Published:** 2018-10-10

**Authors:** Benjamin U. Phillips, Laura Lopez-Cruz, Lisa M. Saksida, Timothy J. Bussey

**Affiliations:** 1 Department of Psychology and MRC/Wellcome Trust Behavioural and Clinical Neuroscience Institute, University of Cambridge, Downing Street, Cambridge, CB2 3EB UK; 2 Molecular Medicine Research Group, Robarts Research Institute & Department of Physiology and Pharmacology, Schulich School of Medicine & Dentistry, Western University, London, ON Canada; 3 The Brain and Mind Institute, Western University, London, ON Canada

**Keywords:** Extinction, Operant, Schedules of reinforcement, Learning, Dopamine, Serotonin, Touchscreen

## Abstract

This review is concerned with methods for assessing the processing of unrewarded responses in experimental animals and the mechanisms underlying performance of these tasks. A number of clinical populations, including Parkinson’s disease, depression, compulsive disorders, and schizophrenia demonstrate either abnormal processing or learning from non-rewarded responses in laboratory-based reinforcement learning tasks. These effects are hypothesized to result from disturbances in modulatory neurotransmitter systems, including dopamine and serotonin. Parallel work in experimental animals has revealed consistent behavioral patterns associated with non-reward and, consistent with the human literature, modulatory roles for specific neurotransmitters. Classical tests involving an important reward omission component include appetitive extinction, ratio schedules of responding, reversal learning, and delay and probability discounting procedures. In addition, innovative behavioral tests have recently been developed leverage probabilistic feedback to specifically assay accommodation of, and learning from, non-rewarded responses. These procedures will be described and reviewed with discussion of the behavioral and neural determinants of performance. A final section focusses specifically on the benefits of trial-by-trial analysis of responding during such tasks, and the implications of such analyses for the translation of findings to clinical studies.

## Deficits in reward omission processing in clinical populations

Appropriate processing of positive and negative outcomes is a requirement for adaptive reinforcement learning, broadly defined as the ability to select actions in a manner that maximizes positive outcomes (wins) and minimizes negative outcomes (losses) (Sutton and Barto 1998). Abnormal sensitivity to wins and losses is a central feature of a number of psychopathological conditions that are characterized by a failure to learn about and respond adequately to outcomes or shifts in environmental contingencies. Importantly, these changes in sensitivity to wins and losses are thought to result in significant consequences for functional outcomes in these conditions, including enduring reductions in quality of life in both major depressive disorder (MDD) (Victoria et al. 2018) and schizophrenia (Mueser et al. 1991; Dowd et al. 2016). Despite this, these deficits in function have not been fully characterized in all psychopathologies and much is still unknown regarding the mechanistic basis of these disruptions. Reward processing can be assessed in clinical populations via a number of procedures. Amongst these, reversal learning, which requires subjects to accommodate a contingency shift (a change in outcomes associated with specific responses), and probabilistic reinforcement learning, which requires subjects to maximize reward in the face of a set of uncertain outcomes, are highly prevalent and have revealed distinctive patterns of performance impairments across distinct clinical populations, with patient groups typically failing to accommodate shifts in task contingencies or adapt appropriately to wins and losses (Elliott et al. 1997; Frank et al. 2004; Dowd et al. 2016). In recent years, a number of closely related procedures have been developed and validated for use in experimental animals, thus providing a methodological framework for translational study of reinforcement-related processes.

As characterization of clinical populations on reinforcement learning tasks has increased, it has become increasingly evident that balancing learning from reward and non-reward and reacting with appropriate behavioral responses is a common disturbance across disorders. Moreover, many common procedures that are used to characterize clinical populations comprise responses that are non-rewarded, either inevitably due to task design (e.g., ratio schedules, stochastically reinforced tasks) or because subjects typically exhibit sub-optimal patterns of responding (e.g., discounting procedures, reversal learning). For example, progressive ratio schedules of reinforcement, which are traditionally used almost exclusively in experimental animals, have recently been used to assess effortful motivation in schizophrenia (Strauss et al. 2016; Bismark et al. 2018) and anorexia nervosa patients (Schebendach et al. 2007).

In a similar vein, cognitive flexibility has been assessed in a number of clinical populations using reversal learning procedures. In reversal learning, subjects must accommodate a shift in task contingencies whereby a previously rewarded response becomes non-rewarded and vice versa. Patients often display “perseverative” deficits, in which responses are persistently emitted at a previously rewarded but newly non-rewarded response option (Miller 1990; Chamberlain and Sahakian 2006; Cools et al. 2007; Murray et al. 2008). These deficits may be at least partially mediated by a failure to appropriately process reward omission and reflect the central nature of reinforcement-related executive dysfunction across a broad range of psychopathologies. In perhaps the clearest clinical example, MDD, which is known to possess a serotonin (5-hydroxytryptamine; 5-HT) dysregulation component, is characterized by both hyposensitivity to rewarding outcomes and hypersensitivity to omission of reward. This is as measured by non-optimal response switching in probabilistic learning tasks in which MDD patients tend to switch response strategy following a loss, even if their previous strategy was optimal (Murphy et al. 2003). This abnormal responsivity has been shown to correlate with reduced activity in the dorsomedial and ventrolateral prefrontal cortices, and increased activity in the amygdala in unmedicated MDD patients (Taylor Tavares et al. 2008). Moreover, the identified pattern of deficits is consistent with both anhedonia and hyperactive emotional responsivity to negative outcomes and predicts disease outcomes (Vrieze et al. 2013), suggesting that imbalances in reward processing in MDD may play a central role in the maintenance of low mood in this disorder. In addition to 5-HT, other neurotransmitters are implicated in this set of behavioral functions. For example, a distinguishable set of deficits related to processing non-reward has been reported in Parkinson’s disease, in which patients performing probabilistic discrimination tasks demonstrate heightened learning from reward omission as compared to rewarding outcomes (Frank et al. 2004). This abnormality is dependent on treatment regimen, as patients taking levodopa show heightened sensitivity to reward and generally intact learning from reward omission, a set of findings in general agreement with a model that emphasizes the importance of dopamine (DA) levels in reinforcement learning feedback sensitivity.

Choice discounting of a preferred reward has also been assessed in clinical patients using probabilistic discounting and delay discounting. In typical discounting procedures, an increasing cost, such as an escalating delay, or reduction in probability, is systematically imposed on access to a preferred reward. Subject choices typically shift from selecting the preferred reward to a less preferred but free reward as the cost increases. Pathological gamblers have been characterized on both probability (Miedl et al. 2012) and delay discounting (Madden et al. 2011; Wiehler et al. 2015), with the results tending to indicate that patients suffering from this condition both select more risky choices and discount future rewards more steeply. Moreover, a large number of clinical conditions, including Parkinson’s disease (Housden et al. 2010), frontotemporal dementia (Bertoux et al. 2015), and major depressive disorder (Pulcu et al. 2014), are also characterized by reduced tolerance of delayed reward. Imaging studies suggest that brain regions including the lateral prefrontal cortex, posterior parietal cortex (McClure et al. 2004), and inferior frontal gyrus (Lin et al. 2015) are involved in the valuation of delayed rewards. Additionally, a number of regions, including the ventral anterior cingulate cortex (vACC) (Kruse et al. 2017) and medial orbitofrontal cortex (mOFC) (Finger et al. 2008), have been implicated in human appetitive extinction learning, suggesting that a broad network of structures are involved in processing reward omission in humans.

Taken as a whole, the extant body of clinical evidence suggests that deficits in translationally viable tasks comprising a substantial reward omission component are present and distinguishable across a number of psychopathological conditions. Thus, this research area represents a valuable opportunity for parallel study of these processes in experimental animals. Moreover, whilst such patient deficits are well characterized and the available evidence suggests that they play a central role in the development and maintenance of psychopathology, the systems that mediate abnormal responsivity to reward omission are not yet fully understood and specific targets for novel treatments remain largely elusive. To facilitate understanding of the psychological and neural mechanisms that govern reward omission processing, it is critical to carry out preclinical studies in experimental animals, ideally using procedures that measure either identical or closely related psychological processes. As feedback integration and reward omission processing are clearly disturbed in numerous psychopathological conditions, the aim of this review is to consider the preclinical application of operant methods that involve an explicit reward omission component, including single-contingency appetitive extinction (where only one response option is available), ratio schedules, reinforcement learning tasks, and discounting procedures. Both procedural considerations and the neural systems implicated in the performance of these tasks are discussed in the context of facilitating understanding of the behavioral methodology. We suggest that applying multiple tasks characterized by reward omission to the same experimental question can greatly facilitate understanding of the change in behavioral state resulting from a defined manipulation. Additionally, this review aims to examine the potential for cross-species translation of results obtained on these tasks (in the context of trial-by-trial analysis of performance (Daw 2011), and touchscreen operant testing (Bussey et al. 2008) is considered.

## Behavioral procedures involving reward omission

### Single contingency procedures: extinction and ratio schedules

Single contingency procedures—as opposed to choice procedures, considered below—involve only one possible response. To assess responding in the face of non-reward in such procedures, reward is omitted following a response. Two widely used approaches of this type are appetitive instrumental extinction schedules, and ratio schedules. In an appetitive extinction procedure, a previously rewarded response becomes abruptly non-rewarded and extinction learning is indicated by discontinued responding. Persistent responding, relative to controls, in the absence of reward is taken to indicate an extinction impairment (Balleine and Dickinson 2000). In addition to the number of responses emitted on extinction sessions, the rate of responding can be measured to generate an additional index of response to non-reward. These schedules have been used to characterize extinction learning in a number of diverse rodent models, including models of fragile × syndrome (Sidorov et al. 2014), NMDA receptor subunit dysfunction (Brigman et al. 2008), and deletion of postsynaptic density protein 95 (Horner et al. 2017).

A number of methodological considerations should be taken into account when applying extinction learning procedures in experimental animals. For example, multiple distinct mechanisms potentially contribute to performance on appetitive extinction schedules, including response suppression, instrumental learning, Pavlovian processes, and detection of non-rewarded responses (Bouton 2004). The reduction in responding under appetitive extinction schedules following reinforcer devaluation, an experimentally induced degradation of reinforcer value, can also depend on reinforcer properties as demonstrated elegantly by Adams and Dickinson (1981). In this study, a food reinforcer was paired with an injection of lithium chloride, thus inducing a conditioned food aversion, and was used in combination with instrumental extinction to reveal the mechanisms of reinforcer relationship to extinction of responding. It was shown that conditioned aversion to the reinforcer attenuated responding at extinction relative to controls, suggesting that reinforcement is represented in the associative structure even when not present within the schedule structure (Adams and Dickinson 1981). Thus, reinforcer valuation effects play a contributory role in extinction schedule performance, even when the reinforcer itself is absent. Thus, researchers should carefully consider the choice of reward used in studies investigating extinction learning under an intended experimental manipulation, as differences in reward processing during acquisition may spuriously affect extinction schedule performance.

Another methodological consideration in the context of extinction procedures is that previous instrumental experience can modulate performance at extinction. For example, animals previously exposed to partially reinforced schedules exhibit enhanced resistance to extinction as compared to animals exposed to a continuously reinforced schedule, an effect termed the partial reinforcement extinction effect (PREE) (Weiner et al. 1985; Bouton et al. 2014). This effect has been shown to depend on dopamine (DA), as potentiation of DAergic neurotransmission with d-amphetamine administration further increases PREE (Weiner et al. 1985). Conversely, DA D2 receptor blockade exerts effects that are partially overlapping with, but not identical to, extinction in animals working on reinforced schedules (Wise et al. 1978; Salamone 1986). Taken together, this evidence suggests that response to reward omission on appetitive instrumental extinction schedules is dependent on exposure to previous schedule, previously encoded incentive value of the reward and DA dynamics. In particular, these DA-dependent effects on extinction responding represent an important consideration in the context of characterization of instrumental extinction learning in genetically modified models with a reasonable probability of DA dysregulation (e.g., mouse models of Parkinson’s disease or schizophrenia).

In contrast with extinction schedules, ratio schedules require emission of a set number of responses in order to gain access to a reward (Sidman and Stebbins 1954; Hodos 1961). Contingencies in ratio schedule testing can be arranged in a number of ways and are typically designed to probe aspects of reward-related behavior. In progressive ratio (PR) schedules, the number of responses (i.e., lever presses, nose-spokes or touches) required to obtain a single reinforcer increases progressively during the session according to a defined ramp. The final ratio completed in a session (referred to as the “breakpoint”) is frequently interpreted as an operational measure of reward value (Hodos 1961) or animal effort capacity (Aberman et al. 1998). However, PR schedules may also probe parallel psychological processes including extinction learning, reward expectancy, and tolerance of unrewarded delays (Ward et al. 2011). In particular, as challenging PR schedules are characterized by eventual large runs of non-rewarded responses at high work requirements, the results may reflect a substantial appetitive extinction component, rather than motivation per se (Ward et al. 2011). Researchers may carry out an extinction schedule control in conjunction with their intended manipulation on PR to assess this possibility.

Moreover, there is substantial evidence that PR and extinction do not assess fully overlapping processes. For example, modulating the DA system affects effort in isolation in other tasks, with systemic administration of DA antagonists and agonists resulting in bi-directional modulation of performance. For example, DA antagonists (i.e., raclopride, haloperidol, and etriclopride) decrease lever presses for a preferred reinforcer (i.e., sucrose pellets) in PR schedules and also increase consumption of freely available chow in fixed ratio (FR) or PR/free chow concurrent choice, suggesting that a number of the effects observed following DAergic manipulations on PR may be attributable to effort processes rather than reward omission processing alone (Farrar et al. 2010; Nunes et al. 2013; Randall et al. 2012; Heath et al. 2015). In addition, there are experimental dissociations between ratio and extinction schedule performance. For example, heterozygous and homozygous DA transporter knockout (DAT-KO) mice show neither higher breakpoints in PR nor higher responses on an FR schedule compared with their wild-type (WT) counterparts (Hironaka et al. 2004). However, during extinction, homozygous DAT-KO mice were more resistant to extinction than WT and heterozygous DAT-KO mice (Hironaka et al. 2004).

Thus, whilst the potential effects of reward omission and consequent extinction processes should be carefully considered in the context of the performance of PR schedules, there is also a large body of experimental evidence suggesting that PR depends on other processes including effort allocation. Testing both PR and extinction procedures may help disentangle the contribution of different psychological processes to task performance. Further approaches to delineating the contribution of different processes to PR performance, including extinction learning and effort, are discussed in the third section of this review. Overall, instrumental extinction performance comprises a large reward omission processing component, but performance also depends on prior reinforcer valuation processes and DA-dependent processes, including previous instrumental contingency experience. Whilst PR performance may depend partially on extinction learning, there is also a large body of evidence suggesting the involvement of effort processes in the determination of responding.

In addition to studies that have focused on general neuromodulatory control of reward omission processing in single contingency procedures, a large body of previous literature has studied the neural circuitry involved in this set of behaviors, especially with respect to instrumental habits. Given that habits are operationally defined as outcome insensitivity (Balleine and Dickinson 1998; Robbins and Costa 2017), these investigations are of obvious relevance to the systems involved in processing reward omission. This body of work has revealed the involvement of a broad network of cortical and sub-cortical structures in the support of habitual responding. Within these broad networks, one particularly influential model of the transition from initial goal-directed to eventual habitual responding at the neural level suggests that initial goal-directed responding is dorsomedial striatum (DMS) dependent but later shifts to dorsolateral striatum (DLS) dependency as habitual processes come to control responding (Corbit et al. 2012). Some studies in humans support this view that this region is critical for control of habitual behavior (Tricomi et al. 2009), whilst also implicating a reduction in activity of the ventromedial prefrontal cortex (vmPFC) in habitual responding (de Wit et al. 2009). With respect to the precise striatal micro-circuitry mediating habits in the DLS, a recent study demonstrated that the activity of fast-spiking interneurons located in this region is closely linked with food-reinforced habitual behavior (O’Hare et al. 2017). In this study, it was shown that silencing this population of interneurons via chemogenetics blocks the behavioral expression of a previously acquired habitual response. In other words, neuronal inhibition targeted at this specific population of DLS interneurons caused mice that had acquired an instrumental habit to behave in a goal-directed manner, perhaps suggesting that these neurons are specifically involved in determining responsivity to rewarding outcomes. Other midbrain systems are also known to encode both habitual responding and reward omission processes. For example, building on the highly influential finding that DA-releasing neurons in the ventral tegmental area (VTA) and substantia nigra encode “reward prediction errors” (i.e., the discrepancy between expected and actual outcomes) (Schultz et al. 1997), Tobler and colleagues later demonstrated that these cells are also predictive of reward omission and behave in a manner consistent with a negative reward prediction error (Tobler et al. 2003). Moreover, a recent study by Verharen and colleagues utilizing fiber photometry and chemogenetics suggested that processing of DA “dips” in the nucleus accumbens encodes learning from losses, further underlining the involvement of this neurotransmitter in reward omission processing (Verharen et al. 2018).

### Choice procedures: reversal learning and probabilistic reinforcement learning procedures

Reversal learning procedures typically require subjects to learn to discriminate between rewarded and non-rewarded responses, before the contingencies are switched so that the previously optimal selection becomes non-optimal and vice versa. At the point of reversal, subjects will typically continue to respond at the previous location for an extended period, a pattern of responding termed perseverative. Whilst reversal learning is designed to assess cognitive flexibility, this term describes a complex construct with multiple constituent contributory psychological components, including response inhibition, attentional processing, reinforcement learning, and reward sensitivity (Nilsson et al. 2015). Critically, in deterministic reversal learning, the previous always-rewarded stimulus (S+) essentially shifts from maintenance under a continuous reinforcement schedule to an extinction schedule, thus raising the possibility that reversal learning performance in the perseverative phase can be attributed to reward omission sensitivity. However, experimental evidence suggests that reversal learning and extinction learning, though perhaps overlapping, are dissociable processes. For example, as pointed out by Horner and colleagues, there is evidence dissociating performance of these procedures (Horner et al. 2017). For example, whilst Grin2a^−/−^ mice are impaired on reversal learning but not extinction (Brigman et al. 2008), Gria1^−/−^ mice demonstrate the opposite pattern of impairments (Barkus et al. 2012). This suggests that, whilst reversal learning deficits can be attributable to loss of sensitivity to extinction, other mechanisms can also mediate impairments.

To perform reversal learning optimally, it is plainly advantageous to not only learn from the absence of reward following a response at the previous S+, but also from reward at the newly rewarded S+. Thus, whilst non-reward processing is an important factor in reversal learning, perhaps particularly in the early stages of a reversal, learning from positive feedback will likely come to predominate toward the end of reversal learning (Phillips et al. 2018). This model of performance at different stages of reversal is consistent with the density of outcomes that is experienced at these stages whereby early reversal is characterized by low performance levels and higher losses and late reversal by high performance levels and higher wins. Such patterns of learning at different phases of both discrimination and reversal learning may also reflect the relative contributions of goal-directed and habitual processes to choice behavior (Graybeal et al. 2011; Brigman et al. 2013; DePoy et al. 2013; Izquierdo and Jentsch 2012; Bergstrom et al. 2018). A number of behavioral probes and manipulations are available to test the balance of these mechanisms, with some results obtained using these procedures suggesting that a number of manipulations that affect reversal learning performance exert their effects by altering sensitivity to reward omission. Methodological approaches for dissociating these contributions will be considered below in the context of studies focused on the neural basis of reversal learning.

Much research has been directed toward the role of 5-HT in the performance of reversal learning tasks. Landmark studies in the marmoset demonstrate that orbitofrontal cortex 5-HT depletions impair reversal learning by increasing the number of perseverative responses emitted in the early phases of reversal (Clarke et al. 2004). This deficit is highly specific, as performance of an extradimensional shifting task, in which subjects have to adapt to discriminating another pair of stimuli based on a different rule, was unimpaired (Clarke et al. 2005). Additionally, the deficit was further investigated using stimulus replacement procedures, in which either the previously rewarded or non-rewarded stimulus is replaced with a novel stimulus. These procedures are designed to determine whether a reversal learning deficit is attributable to stimulus perseveration or learned avoidance of the previous S−. The observed pattern of responding under these manipulations revealed a specific failure to disengage from responding at the previously rewarded stimulus, perhaps suggesting a specific non-reward omission processing deficit in orbitofrontal cortex (OFC) 5-HT depleted subjects (Clarke et al. 2007).

A large body of additional evidence supports a key function for 5-HT in reversal learning, at least partially through influencing the processing of non-rewarded responses. In rodents, treatment with selective serotonin reuptake inhibitors (SSRI) and transgenic SERT (5-HT transporter) inactivation improves performance on reversal learning tasks (Bari et al. 2010; Brigman et al. 2010). At the level of neural circuitry, a recent study combined SERT-cre transgenic mice with fiber photometry to image the activity of dorsal raphe nucleus (DRN) neurons during a reversal learning task (Matias et al. 2017). The results suggest that DRN 5-HT neurons encode trial-by-trial prediction errors in a positive/negative outcome-independent fashion (termed “unsigned” prediction errors). Thus, the authors suggest that DRN 5-HT may have non-specific involvement in processing worse-than-expected rewards by modulating attention or general learning capacity, providing a potential neural substrate for reward omission in the 5-HT system.

In addition to subcortical 5-HT systems, other circuitry has also been shown to be involved in encoding multi-contingency task outcomes during procedures characterized by frequent reward omission. For example, a recent study, utilizing an attentional set-shifting procedure in which rats had to flexibly adapt to discriminate between different reward-predictive features, found that a set of dorsomedial prefrontal cortex (dmPFC) neurons reliably predicted task outcome not only prior to outcome presentation but also post-trial outcome encoding (Del Arco et al. 2017). The role of the PFC is further emphasized by multi-unit recordings carried out in rats performing a gambling task, in which subjects choose between multiple options with different probabilities of more or less desirable outcomes, demonstrating that poor performance in a model of maternal separation was correlated with a loss of synchrony between the anterior cingulate cortex and amygdala (Cao et al. 2016). More broadly, studies in animals carrying out reversal learning procedures have implicated a broad network of structures in encoding outcomes on a trial-by-trial basis including the dorsal raphe nucleus (Barlow et al. 2015; Matias et al. 2017), striatal regions (Klanker et al. 2015), and frontal cortices (Marquardt et al. 2017), suggesting that the neural encoding of outcome signaling is broadly represented in both cortical and sub-cortical circuitries.

In similar approaches, a number of studies have sought to determine the 5-HT subreceptor-specific mechanisms involved in reversal learning performance. The available evidence indicates a central role for 5-HT2C receptors in mediating the influence of 5-HT in processing non-rewarded responses in reversal learning tasks. Specifically, a number of studies have demonstrated that antagonism of the 5-HT2C receptor facilitates reversal learning (Boulougouris et al. 2008), and this effect has been localized both temporally, to the early phases of reversal, and anatomically, to the lateral orbitofrontal cortex (Alsiö et al. 2015). Again, specific effects on early reversal performance are perhaps indicative of alterations in the processing of reward omission. To formally assess the contribution of positive and negative feedback learning to discrimination and reversal performance, a recently developed version of a stimulus-based visual discrimination task leverages a third probabilistically reinforced stimulus to assess learning from positive and negative feedback (Nilsson et al. 2015; Phillips et al. 2018). In the valence-probe visual discrimination (VPVD) task, deterministically reinforced S+ and S− stimuli are occasionally presented in conjunction with an S50 which is reinforced on 50% of the trials. By comparing performance on S+ vs S50 and S50 vs S− trials, it is possible to assess to which stimulus more learning has accrued, thus determining whether the subject has a bias toward learning from reward or omission of reward. The partially reinforced discrimination trials can be presented either during initial discrimination or following a reversal, enabling the contribution of positive and negative feedback learning to these distinct probe trials to be assessed. The task is designed to assess learning from wins and losses in a similar way to multiple procedures available for application in humans. Notable examples include the probabilistic and transitive selection tasks used to characterize reinforcement learning biases in Parkinson’s disease (Frank et al. 2004), Schizophrenia (Waltz et al. 2007), and depression patients (Chase et al. 2010). Additional related tasks used in humans include probabilistic reversal learning tasks that are designed to assess immediate responses to positive and negative feedback (Evers et al. 2005) and reinforcement learning tasks intended to dissociate model based from model free learning (Gläscher et al. 2010). A common structural theme amongst these procedures is that subjects are required to emit responses at (often visually complex) stimuli that are each associated with a probability of reward. Thus, in adopting the same basic features, VPVD may represent a viable translational tool for assessing similar biases in reinforcement learning function in rodent models.

In addition to this potential for translational study, VPVD has already been used to evaluate a potential serotonergic antidepressant in mice (Phillips et al. 2018). In this study, it was found that the 5-HT2C receptor antagonist, SB 242084, impaired discrimination learning of the standard S+ > S− trials. This effect was particularly pronounced in the late sessions, where the performance level of vehicle-treated control animals is high. In terms of the impact of positive and negative feedback on learning as assayed by the additional trial types, 5-HT2C antagonism appeared to shift the balance toward learning from negative feedback. Subsequent experiments employing a spatial probabilistic reversal learning procedure provided further support for this finding, as the same manipulation resulted in a reduction in “win-stay” proportions, an operational index of positive feedback sensitivity.

**Fig. 1:**
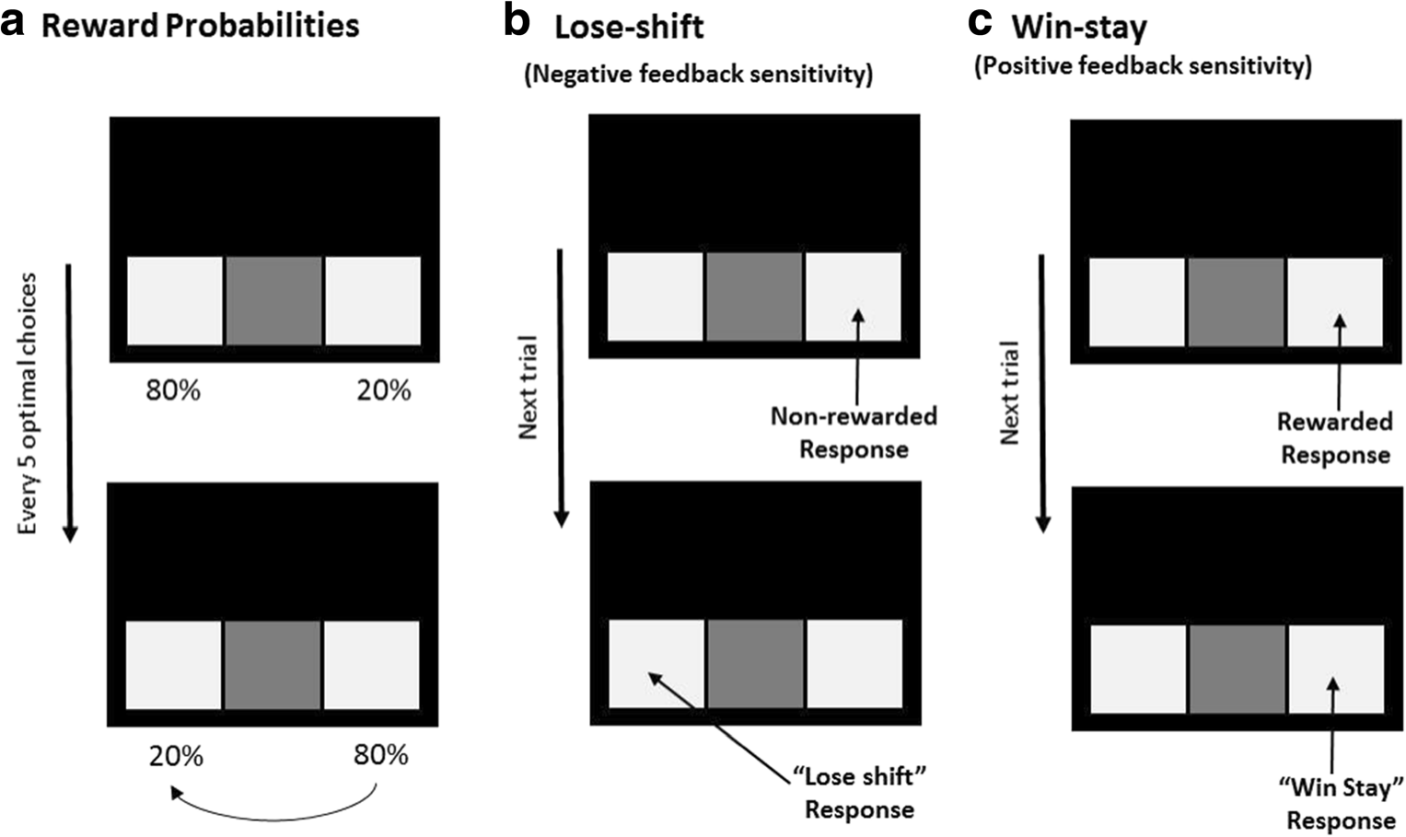
Schematic of a typical rodent spatial probabilistic reversal learning procedure. Rewarding outcomes are stochastically delivered following responses at both spatial locations. Lose-shift is defined as a selection of the alternate spatial location following a non-rewarded trial and is taken as a measure of negative feedback sensitivity. Conversely, win-stay is defined as a selection at the same location following a rewarded trial and is taken as an index of positive feedback sensitivity

In addition to deterministic discrimination and reversal tasks, the role of non-rewarded responses in performance of tasks assaying cognitive flexibility and reinforcement learning is also demonstrated by probabilistic reinforcement learning tasks in humans (Elliott et al. 1997) and experimental animals (Bari et al. 2010) (Fig. 1). In these procedures, responses are stochastically reinforced so that optimal choices are occasionally non-reinforced and vice versa. Contingencies are typically arranged so that overall, approximately 20% of trials are spuriously reinforced. Thus, a requirement for optimal responding in such tasks is that subjects continue to response at the highly reinforced stimulus even following spurious reward omission. Sensitivity to reward and reward omission is measured by analyzing the proportion of trials on which a subject persists at the same stimulus following a rewarded response (win-stay) or responds at the alternative stimulus following a non-rewarded response (lose-shift).

A consistent finding using such procedures is that lose-shift proportions are substantially higher than win-shift proportions, indicating that experimental animals are in general highly sensitive to omission of reward on a trial-by-trial basis (Bari et al. 2010; Ineichen et al. 2012). These proportions are sensitive to a number of manipulations including modulation of 5-HT and glutamate systems (Bari et al. 2010; Rychlik et al. 2016). Specifically, 5-HT appears to be particularly implicated in encoding win-stay lose-shift proportions. In rats, the selective serotonin reuptake inhibitor (SSRI) citalopram exerts dose-dependent effects on accommodation of spurious reward omission (Bari et al. 2010). A recent study suggests that these effects may at least be partially attributable to activity at the 5-HT2C receptor, as systemic agonism of this receptor in mice recapitulates this reduction in lose-shift proportions (Phillips et al. 2018). Taken together, these data suggest that the 5-HT2C receptor is a likely target for the capacity of 5-HT to process reward omission in probabilistic tasks.

### Delay and probability discounting procedures

**Fig. 2:**
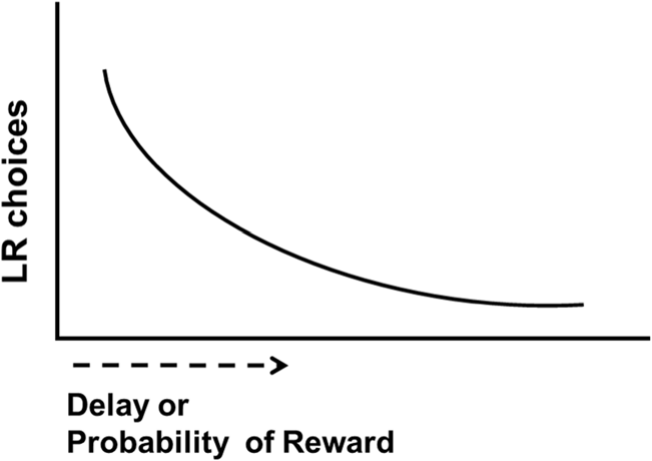
Typical hyperbolic discounting curve observed as a result of discounting procedures. Raw number or percentage choices resulting in the large reward decrease as delay to large reward increases or probability of large reward decreases

Discounting refers to the reduction in value of a preferred option when it becomes associated with a cost such as delay, uncertainty, or effort (Cardinal et al. 2000; Bickel 2015). In perhaps the simplest version of delay discounting, an incrementally increasing delay is imposed between choice and reward delivery. A characteristic discounting choice curve follows a hyperbolic function, reflecting the iterative devaluation of the large reward as a function of delay (Fig. 2). In a similar approach, decision-making under risk can be assessed by probabilistic discounting, in which the probability that a preferred large reward is delivered is systematically reduced across a session (Ghods-Sharifi et al. 2009; Abela and Chudasama 2013). For example, the probability of large reward delivery may begin at 100% and then reduce by 25% across subsequent trial blocks until the probability of large reward is small.

Despite superficial similarities, it is widely recognized that delayed and probabilistic reinforcement recruit distinct neural and psychological processes, as they display differential sensitivity to a number of manipulations (Cardinal 2006). Moreover, since probabilistic discounting is the only procedure amongst these examples in which the outcome is unpredictable, it can be methodologically leveraged to isolate processes associated with reward omission decision-making by comparing results obtained with this procedure with results obtained under delay discounting. In this vein, some studies have utilized delay and probabilistic discounting in parallel to reveal the specific neural mechanisms involved in reward uncertainty and reward delay, thus isolating mechanisms unique to reward omission within an otherwise consistent framework (Yates et al. 2015; Yates et al. 2018).

However, accurate interpretation of results acquired from discounting procedures requires a number of considerations. For example, a potential alternative explanation of delay discounting data is that the response becomes uncoupled from the rewarding outcome as the delay increases. This shift in contingencies at the large reward location could result in a reduction in associability between a response at the large reward response contingency and the delivery of the large reward itself. Thus, in the same sense that PR schedules could potentially be characterized as reflective of extinction processes (because the time between the initiation of a bout of responding and the reward deliver increases), delay discounting could also possess an extinction component. Experimental findings suggest that delay discounting does potentially comprise an extinction component. For example, rats may exhibit reduced preference for the large reward choice during the very first session in which delays are presented (Cardinal et al. 2000). However, it has been demonstrated that reward omission only partially resembles a typical discounting function in well-trained animals, suggesting that extinction learning likely cannot fully account for typical discounting-related preference reduction (Cardinal et al. 2000).

Another set of important considerations regarding the application of delay discounting procedures has been highlighted by analysis of the effects of DAergic manipulations on choice behavior. Specifically, the effects of d-amphetamine on choice behavior are equivocal, with studies reporting both decreased (Evenden and Ryan 1996; Helms et al. 2006) and increased (Krebs and Anderson 2012) large, delayed reward preference. Critically, the effects of this compound on choice behavior can be reversed by reversing the delay presentation order, perhaps suggesting that the effects of amphetamine under baseline conditions reflect a tendency to perseveratively repeat patterns of choices emitted early in the session (Tanno et al. 2014). Alternatively, these results may reflect the tendency of d-amphetamine to accentuate the effects of conditioned reinforcers (Taylor and Robbins 1984; Cardinal et al. 2000), or control timing perception, a set of cognitive processes in which DA is known to play a role (Meck 1983; Soares et al. 2016). Thus, discounting procedures undoubtedly recruit a large number of complex mechanisms and processes that could be examined by modification of discounting procedures to probe alternative psychological explanations. For example, researchers may consider reversing the order of presentation of trial bins on some probe sessions, carrying out sessions run in extinction and closely examine other parameters (e.g., the presence or absence of cues during task performance).

In comparison with delay discounting, the underlying neural and pharmacological mechanisms underlying probabilistic discounting are less well studied. However, a number of investigations have sought to determine the neuropharmacological basis of probabilistic discounting. The available evidence indicates an important role for DA, with activation of both D1 and D2 receptors increasing the proportion of choices emitted for a larger but riskier reward (St Onge and Floresco 2009). In addition to DA manipulations, the involvement of defined neural structures involved in probabilistic discounting has been studied in rodent models. These studies have demonstrated the involvement of the nucleus accumbens (Stopper and Floresco 2011), amygdala (Jenni et al. 2017), and OFC (Abela and Chudasama 2013). In one example, Abela and colleagues (Abela and Chudasama 2013) investigated the effects of OFC and ventral hippocampus lesions in rats performing touchscreen-based delay and probabilistic discounting. It was found that, compared to sham-lesioned controls, OFC-lesioned rats tended to show a reduced tolerance for risk but normal tolerance of delay. However, this pattern of performance was reversed in ventral hippocampus lesioned rats, which exhibited reduced tolerance of delay but normal tolerance of risk. These results suggest an important role for the OFC in encoding reward omission in this task framework, thus demonstrating the utility of comparison between delay and probabilistic discounting following defined experimental interventions.

From a methodological perspective, the test species is an important consideration in the context of the application of discounting tasks. There are few published studies that have successfully demonstrated the ability of mice to distinguish between large and small rewards in the context of discounting procedures. However, some authors have reported successful quantity discrimination in the context of “adjusting-amounts” procedures in which the delays are held constant and the amount of reward delivered parametrically varied (Mitchell 2014). In our laboratory, we have found that mice are capable of discriminating different quantities of a palatable liquid reward (strawberry milkshake) to a high level and that this preference supports robust delay and effort discounting (Phillips et al. unpublished data; Lopez-Cruz et al. unpublished data). Moreover, in laboratories employing variants of these tasks with a range of apparatus, rewards, and species, it is important to confirm that subjects are able to both discriminate reward quantities under baseline conditions (i.e., no delay or 100% reward probability) and that this preference is not disrupted by the experimental manipulation.

More broadly, some studies have demonstrated that rodents behaviorally express preferences for rewards of different sweetness/flavor that are of the same quantity (Pardo et al. 2015) and that such preferences may be subject to a high degree of variability between individuals (Sweis et al. 2018). Whether the same processes would be recruited for the performance of discounting tasks leveraging preferences for the same quantity of differentially preferred rewards is an interesting open question.

## Trial-by-trial analysis and cross-species translation

Assessing responses to non-reward in experimental animals arguably necessitates trial-by-trial approaches to data analysis. This is because the usual whole-session measures fail to capture the animal’s immediate responses to changes in the patterns of outcomes. For ratio tasks, one approach to trial-by-trial analysis is to calculate a rate of responding for each trial, the form of which can then be subsequently described by mathematical equations (Killeen 1994; Bradshaw and Killeen 2012). In PR for example, it is typical for rodents to first emit responses rapidly, before declining toward inactivity as the number of trials increases. This decay is known to follow an exponential function. A simple approach is to fit this in a trial-by-trial manner with an equation that captures this decay (Simpson et al. 2011; Phillips et al. 2017). From there, coefficients that represent the predicted peak response rate and decay rate can be extracted and tested for between-group significance. From these, decay rate is hypothesized to reflect reinforcer control over behavior and extinction sensitivity (Ward et al. 2011), whereas peak response rate may reflect initial motivation for the reward or differences in motoric capacity.

For reinforcement learning procedures, a set of recent advances have utilized trial-by-trial modeling to reveal hidden parameters that drive task performance (Daw 2011). These models tend to be based on prediction error learning algorithms that update choice values based on the discrepancy between expected and actual outcomes. From such models, it is possible to extract values for a number of different parameters, including but not limited to learning rates, which can be separated by wins and losses, “stickiness” (the tendency to repeat choices), and inverse temperature (a measure reflective of the sharpness of choice, i.e., the degree to which a subject chooses either in accordance with the perceived value of the available responses). These approaches have already been applied in human (Gläscher et al. 2010), non-human primate (Clarke et al. 2014), and rodent (Verharen et al. 2018) studies, and have been used to investigate the roles of both DA (den Ouden et al. 2013; Eisenegger et al. 2014) and 5-HT (den Ouden et al. 2013; Iigaya et al. 2018) on performance. For example, this class of approaches has shown that rats treated with a stimulant that potentiates DAergic neurotransmission, methamphetamine, exhibit impaired learning from losses (reward omission) on a reversal learning task (Verharen et al. 2018). Additionally, in the general context of decision-making under risk, it has been demonstrated that D2Rs play an important role in encoding trial-by-trial choices when reward omission is possible (Zalocusky et al. 2016). Thus, reinforcement learning modeling is a promising novel direction for investigation of the learning mechanisms related to reward omission across species. However, ensuring equivalent task design as far as possible is an important requirement to fully realize the potential of such approaches so that the extracted parameters can be compared as far as is reasonably possible.

More broadly, in translational terms, it is beneficial to employ tasks with a high degree of translational potential across multiple species, not only to ensure that findings in animals are as translational as possible but also to facilitate back-translation of human findings to preclinical studies. One approach directed toward this end is the operant touchscreen testing system, which has already been used to directly assess the performance of both rodents and humans with comparable genetic mutations on the same cognitive tasks (Nithianantharajah et al. 2013; Nithianantharajah et al. 2015). This approach is advantageous not only because tasks can be applied in very similar ways in multiple species but also because a battery of tasks can be applied using the same stimuli, responses, and reinforcers within the same species (Bussey et al. 2008). Indeed, all the tasks described in this review are available in the touchscreen apparatus, and the internal consistency of this approach may allow for better comparison of the effects of reward omission across multiple tasks. It would in theory be possible to apply a large set of the preclinical tasks described here in the same cohort of experimental animals, thus facilitating more precise comparison of results and a better understanding of the mechanisms involved in different aspects of behavioral responses to reward omission.

## Conclusions

Clinical data suggest that the processing of the omission of reward is a dysregulated process in a number of neuropsychiatric and neurodegenerative conditions including MDD, Parkinson’s disease, obsessive compulsive disorder (OCD), and addiction. A clear example is MDD, in which oversensitivity to reward omission manifests as impairments in reinforcement learning in laboratory tasks and may constitute a central pathological process in the maintenance of low mood in this disorder (Elliott et al. 1997). Failures in appropriate reward omission processing can be hypothesized to result from dysregulation of a number of neural systems. For example, both DA and 5-HT appear to play a particularly important mechanistic role in this domain in both health and disease.

A large number of procedures at the preclinical level feature omission of rewarding outcomes, though the focus and interpretation of many such studies often hinge on response to reward. A good deal of progress has been made in elucidating the neural basis of processing reward omission using such tasks. Some conclusions regarding behavioral effects across preclinical tasks can be drawn. For example, reward omission tends to promote decreased vigor in single contingency tasks whilst tending to promote choice switching in multiple contingency tasks. These baseline tendencies can be affected by manipulation of the neuromodulatory systems involved in encoding these responses, specifically DA and 5-HT. Importantly, it is known that whilst these systems may possess distinct behavioral functions, they closely interact in the context of encoding wins and losses (Daw et al. 2002).

Given the clear importance of reinforcement learning deficits from a clinical perspective, we suggest that researchers continue to develop preclinical procedures specifically designed to assess processing of, and response to, reward omission, and that trial-by-trial analytical techniques are applied to maximize translational potential. Moreover, we suggest the application of multiple types of preclinical procedures to study this set of clinically relevant domains, as patterns of responding are divergent across tasks and afford an unprecedented opportunity to dissect the precise neural circuitry involved in encoding reward omission responses.
